# Double hit of *NEMO* gene in preeclampsia

**DOI:** 10.1371/journal.pone.0180065

**Published:** 2017-06-27

**Authors:** Agata Sakowicz, Tadeusz Pietrucha, Magda Rybak-Krzyszkowska, Hubert Huras, Agnieszka Gach, Bartosz Sakowicz, Mateusz Banaszczyk, Mariusz Grzesiak, Lidia Biesiada

**Affiliations:** 1 Department of Medical Biotechnology, Medical University of Lodz, Lodz, Poland; 2 Department of Obstetrics and Perinatology, University Hospital in Krakow, Krakow, Poland; 3 Departments of Genetic, Polish Mother's Memorial Hospital-Research Institute in Lodz, Lodz, Poland; 4 Department of Microelectronics and Computer Science, Lodz University of Technology, Lodz, Poland; 5 Department of Tumor Biology, Medical University of Lodz, Lodz, Poland; 6 Department of Obstetrics and Gynecology, Polish Mother's Memorial Hospital-Research Institute in Lodz, Lodz, Poland; Xavier Bichat Medical School, INSERM-CNRS - Université Paris Diderot, FRANCE

## Abstract

The precise etiology of preeclampsia is unknown. Family studies indicate that both genetic and environmental factors influence its development. One of these factors is NFkB, whose activation depends on NEMO (NFkB essential modulator. This is the first study to investigate the association between the existence of single nucleotide variant of the *NEMO* gene and the appearance of preeclampsia. A total of 151 women (72 preeclamptic women and 79 controls) and their children were examined. Sanger sequencing was performed to identify variants in the *NEMO* gene in the preeclamptic mothers. The maternal identified variants were then sought in the studied groups of children, and in the maternal and child controls, using RFLP-PCR. Real-time RT-PCR was performed to assess *NEMO* gene expression in maternal blood, umbilical cord blood and placentas. The sequencing process indicated the existence of two different variants in the 3'UTR region of the *NEMO* gene of preeclamptic women (IKBKG:c.*368C>A and IKBKG:c.*402C>T). The simultaneous occurrence of the TT genotype in the mother and the TT genotype in the daughter or a T allele in the son increased the risk of preeclampsia development 2.59 fold. Additionally, we found that the configuration of maternal/fetal genotypes (maternal TT/ daughter TT or maternal TT/son T) of IKBKG:c.*402C/T variant is associated with the level of NEMO gene expression. Our results showed that, the simultaneous occurrence of the maternal TT genotype (IKBKG:c.*402C>T variants) and TT genotype in the daughter or T allele in the son correlates with the level of NEMO gene expression and increases the risk of preeclampsia development. Our observations may offer a new insight into the genetic etiology and pathogenesis of preeclampsia.

## Introduction

Preeclampsia is a phenomenon which appears in 5–8% of pregnancies and represents a serious problem concerning both maternal and infant mortality and morbidity [1,2]. Although the precise etiology of preeclampsia is unknown, it has been suggested that while both genetic and environmental factors influence its development, genetic factors have a greater influence, accounting for over half of preeclampsia liability cases [3–5]. Clinical observations indicate that women born from preeclampsia have a higher risk of the disease during their own pregnancies than those without any such family history [6,7]. The effect of inheritance is much stronger along the maternal than the paternal line: 2.2 vs 1.5 respectively [8]. As the expression of preeclampsia may differ between two pregnancies borne by the same woman, fetal (paternal) genes have also been proposed to play a role; the contribution of fetal genes to preeclampsia development has been estimated to be 20% [3,4]. Inadequate maternal immunological response to the allogenic fetus and abnormal trophoblast invasion into the spiral arterioles is considered the main reasons for maternal hypertension and proteinuria development [9,10].

While a balance exists between Th1 and Th2 responses in healthy, non-pregnant women, a shift towards Th2 is observed during pregnancy. Some studies note that in preeclampsia, the shift towards Th2 probably does not occur or is reversed at very early stages of the disease [9,11]. Preeclamptic pregnancies have been characterized as a maternal pro-inflammatory state with predominant Th1 and Th17 lymphocytes [12]. Additional sources of pro-inflammatory cytokines (IL-1, TNF-α, IL-6, IL-8) in preeclampsia are monocytes and macrophages [1,11]. Moreover, the blood of preeclamptic women is known to contain elevated levels of such other factors as soluble receptor of IL-6 (sIL-6R), soluble fsm-like tyrosine kinase-1 (sFlt-1), soluble form of Fas ligand (sFasL) and NFĸB [1,13,14].

NFĸB is a transcription regulator of over 200 different genes, of which a number encode the proteins elevated in the blood of preeclamptic women [15]. These products e.g. IL-6, TNF-α or Il-1 are not only dependent on NFĸB but have also been implicated in the process of NFĸB activation on the classical pathway [16]. The core element of NFĸB activation on the canonical pathway is the IKK kinase complex (IKKα and IKKβ) and IKKγ, a regulatory subunit known as NEMO or IKBKG.

NEMO functions as a scaffolding protein and links upstream signaling pathways to activation of the IKK complex [17]. Outside of the canonical pathway exists other pathways (non-canonical and atypical) described in the literature. These are responsible for activation of the NFkB factor. However, the canonical pathway is the most common in all tissues and it is related to the response of the cell to TNFα and IL-6, whose elevated levels are observed in the blood of preeclamptic women [18,19].

Although NEMO does not have catalytic properties, NEMO-defective cell lines do not activate NFĸB in response to many stimuli, suggesting that the protein plays a key role in the activation of NFĸB pathway [20]. In a study on mouse keratinocytes, Nenci et al [21] found that a lack of NEMO leads not only to the spontaneous death of the cells, but also triggers the pro-inflammatory expression of mediators in adjacent wild-type cells. The results of this study are in consistency with observations of human skin biopsies of patients with *NEMO* gene defect and suffering from incontigentia pigmenti [22]. Moreover, examinations conducted on cardiomyocytes found that an absence of active NEMO protein results in the inactivation of antioxidant processes, followed by spontaneous pathological tissue remodeling, cell death, fibrosis and contractile dysfunction caused by the subsequent accumulation of free oxygen radicals [15]. Very similar pathomorphological observations have been noted in several studies conducted on placentas derived from preeclamptic pregnancies [23,24]. Moreover, the level of *NEMO* gene expression in placentas from pregnancies complicated by preeclampsia is significantly lower than observed in non-complicated pregnancies. NEMO level may hence be one of the reasons for the abundance of preeclamptic placental apoptosis [25].

In contrast to placentas derived from pregnancies complicated by hypertension and proteinuria, a higher level of *NEMO* gene expression has been observed in the blood of preeclamptic women (according to 1A, 1B, 1C transcripts and all transcripts together—Total NEMO) and their children (according to 1A transcript) [25]. Differences in the expression of the IKKγ gene in the tissues of preeclamptic and healthy women and their children may be related to the existence of some variation in the NEMO coding gene. The observations related to the maternal lineage of inheritance, the differential expression of the disorder in two pregnancies by the same woman described above, and the contribution of fetal (paternal) genes to the development of preeclampsia suggest that this variation may exist both in the maternal and fetal *NEMO* genes.

The purpose of our study was to determine any difference between the sequences of *NEMO* genes derived from preeclamptic women and those from healthy women and their children. We hypothesize that the simultaneous existence of the same variant in the maternal and fetal *NEMO* gene may be associated with a higher risk of preeclampsia development We also would like to check whether the identified variant is associated with the level of *NEMO* gene expression (Total NEMO and 1A, 1B, 1C transcripts) in placentas and in fetal and maternal blood.

## Methods

### Patient selection and data collection

In total, 151 women and their children were enrolled to the study (N = 72) and control groups (N = 79). The study group included pregnant women with symptoms of preeclampsia: maternal blood pressure higher than 140/90 mm Hg (measured twice with an interval of at least six hours) with accompanied proteinuria (> 300 mg per 24 hours or at least 2+ during a single urine test) which developed after the 20^th^ week of gestation. The study group included both patients with early (<34 weeks of pregnancy; N = 27) and late (≥34 weeks of pregnancy; N = 45) preeclampsia.

The remaining qualification criteria for examination were the same for control and study groups: single pregnancy, no hypertension and diabetes mellitus before pregnancy, no gestational diabetes mellitus, no chromosomal aberration in the fetus, maternal body mass index (BMI) before pregnancy ≤30kg/m^2^, and no other chronic maternal disorders. Both groups included only women whose baby was delivered by caesarian section. In the preeclamptic group, all women delivered the baby by caesarian section due to clinical indications. As the level of the active form of NFkB is elevated in placentas during the term of labour and NEMO is an essential regulator of NFkB, we decided to collect the control samples also from women who had delivered their baby by caesarian section. Indications for caesarean delivery in control group were as follows: transverse or breech position of fetus, ophthalmological indications, orthopedic indications, or increased risk of uterine rupture because of previously performed cesarean delivery.

Informed consent was signed by each mother before delivery, and the study protocol was approved by the Medical University of Lodz Ethical Committee.

The whole venous blood was taken from all participants 1–2 hours before the beginning of delivery, whereas the umbilical blood and a fragment of placenta were obtained immediately after birth. The procedure concerning the protection and storage of placenta fragments is described elsewhere [25].

### Isolation of nucleic acids

DNA was isolated from maternal blood and fetal umbilical blood using a commercial kit (Chemagen, Germany). DNA quality and purity were determined spectrophotometrically (NanoDrop; Thermo Fisher Scientific, Grand Island, NY). The obtained DNA was stored at -20°C for further analysis. RNA isolation and transcription into cDNA was described elsewhere [25].

### *NEMO* gene sequencing

*NEMO* gene consist of 10 exons. Exon numbers 1A, 1B, 1C and 2 were sequenced directly from DNA, while a region consisting of 10,065 nucleotides was amplified to sequence exons three to ten. This Long Fragment was sequenced using 500ng of DNA and a pair of primers: For_5'- AGAGACGCCACTAACTTGCC-3' and Rev_5'- CAGAGAACTGCATGGCCTAG-3'. This procedure was necessary due to the avoidance of a *NEMO* pseudogene with the same sequence as the *NEMO* gene from the 3^rd^ to 10^th^ exon. Both genes (*NEMO* and its pseudogene) lie head to head on the X chromosome [22,26].

The sequencing procedure was conducted only in the group of preeclamptic women. The results of the sequencing were compared with reference sequences listed in available databases: ENSEMBL GRCh38.p5 (http://www.ensembl.org/index.html) and Human Genome Browser GRCh38/hg38 (http://www.genome.usc.edu). The pairs of primers used in sequencing processes are given in Table 1.

**Table 1:** Primers used in sequencing processes.

Exon	Primer Forward 5'→3'	Primer Reverse 5'→3'	Length of frag.
1A	GGGGAGTGCCAACATCATCA	TACCTCACACTTGCCCACAC	475bp
1B	CACGCCTCCTCTGGGCTG	GGAGCAGGAACGGGCTGG	631bp
1C	CCAAAGACAGGGTTCCATCC	AGTGACCTCCGTGCTATTCC	455bp
2	TGTAGCCCCAGAACTCATAGGC	GAAACCAAGAGGGAGGGCAG	399bp
3^a^	GCAGAAAGTGAGGGGCATTAGTG	GAGTGTGACGGCTTTTCTGAGTTC	593bp
4^a^	CTAGGGCACCCAGGTTTGG	TCTGCCCCGACGTGTTTAC	406bp
5^a^	CATCAGCTCGCAGTCACAG	CCGACACTTCTCAGCCTTTC	351bp
6^a^	AAGGGGGTAGAGTTGGAAGC	AGGCAAGTCTAAGGCAGGTC	276bp
7^a^	TCAGCATCTCCTCTGTCGTT	GGGCAACAAGAGCAAAAC	344bp
8^a^	GCATTACTCGCCGACGTATC	ATCCGTCTCCTGTGGTCAC	413bp
9^a^	GTGACCACAGGAGACGGAT	CAGAGAGCAACAGGAAGGTC	316bp
10^a^^,^^b^	GCGGCTCCTGGTCTTACA	GTGGTTCGAGCAGACAGAAG	517bp
	CACCTTACGCTTCAGCTGTTG	CAGAGAACTGCATGGCCTAG	634bp

^a^ the template for application of these fragments was 200x diluted the Long Fragment of *NEMO* gene achieved after PCR reaction (procedure was necessary to omit the pseudogene of NEMO gene).

^b^ exon 10 was divided into two fragments

After the first PCR reaction, the products were purified using QIAquick PCR Purification Kit (QIAGEN). Sequencing was conducted in both directions, using a BigDye Terminator Kit v3.1 (Applied Bio Systems, USA).

### PCR-RFLP assay

PCR-RFLP was used to confirm the presence of two variants in NEMO gene among preeclamptic women identified by sequencing. The same method was applied to examine whether these variants were absent in the children of the preeclamptic women, and in the mothers and children in the control group. As the variants identified in the sequencing processes were located in exon 10, it was necessary to amplify the Long Fragment (10,065 nucleotides) of DNA both of children born from preeclampsia and the members of the control group. The following primers were used to search for single nucleotide variants: For_1_5'-CTTCCCCTGGCAGAaCTG-3' and Rev_1_5'- CATGCTCAGTGGTGTCACTTC-3' for detection of the first variant; For_2_5'-CGGACACCGACtCGCCaG-3' and Rev_2_5'- CCATCTACGCCATCGCCCATG-3' of the second one. After amplification, fragments were cut using restriction enzymes. The fragment for the first variants (292bp) was cut by the HaeIII restriction enzyme and the fragment for the second variant (270bp) by AluI.

### Gene expression analyses

The processes of gene expression analysis for Total NEMO, and the 1A, 1B and 1C transcripts of the *IKBKG* gene were conducted according the protocols described elsewhere [25].

### *In-silico* analyses

Secondary mRNA structure predictions were carried out by the RNAfold Online tool http://rna.tbi.univie.ac.at/cgi-bin/RNAfold.cgi. [27,28]. Two different software packages: RegRNA (http://regrna2.mbc.nctu.edu.tw/detection.html) [29] and miRNA_targets (http://mamsap.it.deakin.edu.au/~amitkuma/mirna_targetsnew/sequence.html)[30] were used to study potential microRNA (miRNA) recognition sites in the 3’UTR region. These analyses were conducted to check whether variations in the 3’UTR region may be involved in the posttranscriptional regulation of NEMO mRNA level by modification of the minimum free energy of folding (MFE) of mRNA and/or ligation sites for some miRNAs.

### Statistical analysis

Data analysis was performed using Statistica v12 (StatSoft, Tulsa, OK). The normal distributed clinical and personal characteristics data were compared between groups using Student t test. The Hardy—Weinberg equilibrium was tested in maternal control and fetal-girl control groups. Nominal variable comparisons were performed using Chi^2^ or Yates' corrected Chi^2^ tests. Odds ratios (OR) with 95% Confidence Intervals (95%CI) were calculated where possible. Multivariate analysis was performed with backward stepwise logistic regression on variables found to be significant (p<0.05) in univariate analyses. Nonparametric data we analyzed using the Mann-Whitney U test or the Kruskal-Wallis and Dunn’s *post-hoc* tests. The Haploview software was used to assess the linkage disequilibrium for variations selected on *NEMO* gene [31].

## Results

### Baseline of the study and control populations

A total 151 women and their children were qualified to this study. Collection of this number of cases was possible due to extension of our previous control and study groups which are presented elsewhere [25]. All members of the study group were Caucasian and resident in Poland. No discrepancy in ethnicity which may influence various distribution of genetic markers was found between the study and control groups. The comparisons of the baseline laboratory parameters and characteristic of patients of the study and control groups are summarized in Table 2.

**Table 2:** Comparison of clinical data within the study population.

Clinical data	Preeclamptic group	Controls	P value
	(n = 72)	(n = 79)	
Age of women (years)^a^	30.4±6.0	32.0±4.1	0.052
BMI (kg/m^2^)^a^	26±3.2	25±3.6	<0.01
WBC (10^3^/μl)^a^	10.6±2.4	10.8±2.2	0.585
RBC (10^6^/μl)^a^	4.1±0.5	4.1±0.4	0.405
HB (g/dl)^a^	12.2±1.5	12.4±1.1	0.287
HCT (%)^a^	35.5±3.6	36.2±2.6	0.211
MCV (fl)^a^	86.2±4.1	87.2±5.3	0.202
MCHC (g/dl)^a^	34.4±2.7	33.9±1.6	0.126
PLT (10^3^/μl)^a^	198.1±61.7	224.3±50.6	<0.05
Baby weight (g)^a^	2510±931	3399±459	<0.001
Baby height (cm)^a^	49.0±6.2	54.1±2.9	<0.001
Apgar score—1 minute^a^	8,6±1.5	9,3±0.9	<0.001
Miscarriage n (%)^b^	13 (19%)	12 (15%)	0.609
Baby sex (son) n (%)^b^	46 (64%)	36 (46%)	<0.05
Week of delivery^a^	31.7±2.0 for early PE (n = 27) 37.6±2.1 for late PE (n = 45)	38.5±1.0	<0.0001 <0.01
Primiparas n (%)^b^	55 (76%)	29 (37%)	<0.0001

^a^ The values are presented as mean±standard deviation. For data analysis t-Student test was used;

^b^ For data analysis Chi square test was used

Legend: BMI, body mass index; WBC, white blood cells; RBC, red blood cells; HB, haemoglobin concentration; HCT, haematocrit; MCV, mean corpuscular volume; MCHC, mean corpuscular haemoglobin concentration; PLT, platelets; kg/m^2^, kilograms/meter square; μl, microlitre; g/dl, grams/decilitre; %, percent; fl, fentolitre; g, grams; cm, centimetre; n, number of cases

### Influence of IKBKG gene variations on the risk of preeclampsia development

The sequencing results reveal that two single nucleotide variants exist in the *NEMO* gene of preeclamptic women. Both are localized in the 3'UTR region in the positions IKBKG:c.*368C>A and IKBKG:c.*402C>T. These two variants are in moderate linkage disequilibrium (LD; r^2^ = 0.54; D’ = 0.89)

The IKBKG:c.*402C>T variant is described in the SNP database as number rs782437119 (http://www.ncbi.nlm.nih.gov/projects/SNP/snp_ref.cgi?rs=782437119).

The occurrences of these identified variants were examined in the preeclamptic children group and in the mothers and children control groups. The frequency of the analyzed genotypes for both variants in the maternal controls and in female child controls was consistent with the Hardy-Weinberg equilibrium.

The univariate analyses of IKBKG:c.*368C>A and IKBKG:c.*402C>T variants revealed no statistical difference in the distribution of genotypes between the members of the study group and those of the control group. Similarly, no significant difference was found in the distribution of the A allele of IKBKG:c.*368C>A variant and the T allele of IKBKG:c.*402C>T variant between the children (male and female) in the study and control groups (Table 3).

**Table 3:** Comparison of genotype frequency between mothers of the study and control groups, between daughters of the study and control groups, and a comparison of recessive allele frequency between children of the study and control groups.

Maternal genotype	Study group N = 72	Control group N = 79	p^a^	p HWE^b^
	Number of cases (%)	Number of cases (%)		
IKBKG:c.*368C>A				
CC	2 (3%)	4 (5%)	0.763	0.179
CA	12 (17%)	19 (24%)	0.259	
AA	58 (80%)	56 (71%)	0.166	
IKBKG:c.*402C>T (rs782437119)				
CC	2 (3%)	6 (8%)	0.339	0.119
CT	15 (21%)	22 (28%)	0.315	
TT	55 (76%)	51 (65%)	0.111	
Baby (girls) genotype	Study group N = 26	Control group N = 43	p^a^	p HWE^b^
	Number of cases (%)	Number of cases (%)		
IKBKG:c.*368C>A				
CC	0 (0%)	1 (2%)	0.798	0.534
CA	2 (8%)	8 (19%)	0.371	
AA	24 (93%)	34 (79%)	0.264	
IKBKG:c.*402C>T (rs782437119)				
CC	2 (8%)	1 (2%)	0.653	0.623
CT	4 (15%)	14 (33%)	0.196	
TT	20 (77%)	28 (65%)	0.445	
Recessive baby alleles	Study children group	Control children group	p^a^	
IKBKG:c.*368C>A				
A	92 (94%)	110 (90%)	0.318	
IKBKG:c.*402C>T (rs782437119)				
T	86 (88%)	100 (82%)	0.238	

^a^ For data analyses the Chi^2^ or Yates' corrected Chi^2^ tests were used.

^b^ pHWE—the frequency of analyzed genotypes in control group (mother and daughter) assessed using Hardy-Weinberg equilibrium; p>0.05 the results are consistent with Hardy-Weinberg equilibrium;

Because we hypothesized that predisposition to preeclampsia development may be related to simultaneous occurrence of the same variant in maternal and fetal *NEMO* gene, further analysis was performed to check whether there is a statistical difference between the study and control subgroups consisting of maternal and fetal cases in whose genomes we observed only an AA genotype (mothers and daughters)/A allele (sons) for the IKBKG:c.*368C>A variant or only a TT genotype (mothers and daughters)/T allele (sons) for the IKBKG:c.*402C>T variant. It was found that the co-occurrence of a maternal TT genotype and a TT genotype in the daughter or T allele in the son was significantly more frequent in the preeclamptic group compared to controls (see Table 4).

**Table 4:** Comparison of the simultaneous occurrence of an AA genotype in the mother and her child (AA in her daughter or A allele in her son) (IKBKG:c.*368C>A), and the simultaneous occurrence of a TT genotype in the mother and her child (TT in her daughter, or T allele in her son) (IKBKG:c.*402C>T).

Maternal-fetal variant	Study group N = 72	Control group N = 79	p^a^
	Number of cases (%)	Number of cases (%)	
IKBKG:c.*368C>A			
Maternal AA and child AA (girls) or A (boys)	57 (79%)	55 (70%)	0.179
IKBKG:c.*402C>T (rs782437119)			
Maternal TT and child TT (girls) or T (boys)	53 (74%)	45 (57%)	<0.05

^a^ For data analyses the Chi^2^ was used

The clinical and genetic parameters such as BMI, number of maternal platelets, sex of children and simultaneous occurrence in mother of TT genotype and TT genotype in daughter or T allele in son were included to multivariate logistic regression analysis. It was found that the maternal BMI was associated with a greater risk of preeclampsia (OR = 1.18, 95%Cl 1.06–1.32; p<0.01). Moreover, this analysis revealed that simultaneous occurrence of TT genotype in mother and TT genotype in female child or T allele in male child was related to a higher likelihood of preeclampsia development (OR = 2.59, 95%Cl 1.20–5.57; p<0.05).

### NEMO gene expression level and preeclampsia development

In the present study, we confirm our previous findings that the level of Total NEMO, 1A, 1B, 1C transcripts are significantly elevated in maternal blood (p<0.001) [25]. We also confirm that the level of Total NEMO, 1A, 1B and 1C transcripts are significantly lower in preeclamptic placentas (p<0.01). Our novel finding is that the level of NEMO gene expression is related to umbilical cord blood. The Mann-Whitney U test used in this study, based on almost twice the number of cases than the previous study, found that the 1A, 1B and 1C transcripts are significantly elevated in the preeclamptic umbilical blood (S2 Table, Supporting information).

After the division of preeclamptic women into groups according to the labour term, i.e. before and after the 34th week of pregnancy (late N = 45; early N = 27), that the levels of Total NEMO, 1A, 1B and 1C transcripts in maternal blood are significantly higher in the both preeclamptic subgroups in comparison to controls (p<0.01), as noted in our previous study. The relationship between *NEMO* gene expression and early, late and control groups was found to be similar to that seen in our previous study. Significantly lower *NEMO* gene expression in placental cells was found only between the late preeclamptic group and controls (p<0.01 for Total NEMO, 1A, 1B and 1C transcripts); no significant difference was found between early preeclampsia and control with regard to placental *NEMO* gene expression. A significant finding was that the gene expression of 1A and 1C transcripts in the umbilical cord blood was found to be significantly higher than controls both in late and early preeclampsia (late vs control: 1A transcripts -fold change 1.8 p<0.05; 1C transcripts—fold change 2.4 p<0.05; early vs control: 1A transcripts—fold change 3.7 p<0.001; 1C transcripts—fold change 2.4 p<0.05).

### Effect of IKBKG:c.*402C>T variation on *NEMO* gene expression

It was also found that the simultaneous occurrence of the TT maternal genotype (IKBKG:c.*402C>T) and the TT genotype in the daughter or the T allele in the son influence on the level of *NEMO* gene expression both in blood of mother and her fetus (Table 5).

**Table 5:** Comparison of *NEMO* gene expression level (Total NEMO and 1A, 1B, 1C transcripts) and simultaneous occurrence of TT genotype in the mother and her child (TT genotype in her daughter or T allele in her son).

Transcripts	Mother TT and daughter TT or son T in preeclamptic and control groups		p^a^	Fold change
	*OBSERVED*	*NOT OBSERVED*		
**MOTHERS**				
Total NEMO	2.049	1.058	0.111	1.9
1A	0.536	0.263	<0.05	2.0
1B	0.428	0.204	0.170	2.1
1C	0.362	0.098	0.174	3.7
**CHILDREN**				
Total NEMO	4.702	3.079	<0.05	1.5
1A	1.062	0.677	<0.05	1.6
1B	1.019	0.573	0.108	1.8
1C	1.782	0.515	<0.01	3.5
**PLACENTAS**				
Total NEMO	1.759	1.699	0.429	1.0
1A	0.537	0.576	0.279	0.9
1B	0.376	0.372	0.954	1.0
1C	0.500	0.598	0.843	0.84

The values are presented as median of relative gene expression levels calculated by Pfaffl method.

^a^ P value was calculated using Mann-Withney U-test.

### Effect of IKBKG:c.*402C>T variation on stability of NEMO mRNA

**Fig 1:**
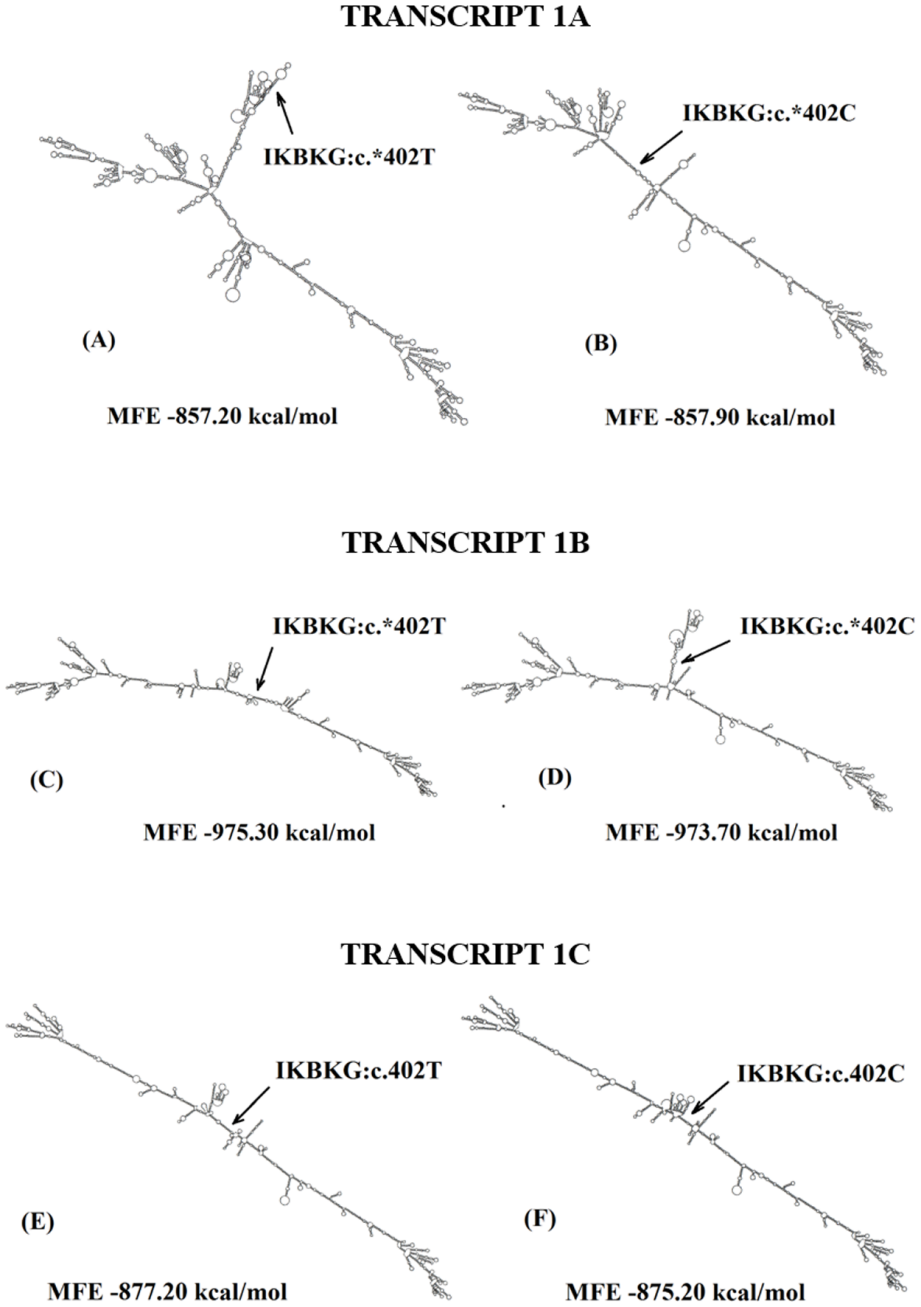
The influence of IKBKG:c.*402C/T variation on the secondary structure of 1A, 1B and 1C transcripts. Analysis was conducted using RNAfold software. (A) transcript 1A with 402T variant; (B) transcript 1A with 402C variant; (C) transcript 1B with 402T variant; (D) transcript 1B 402C variant; (E) transcript 1C with 402T variant; (F) transcript 1C with 402C variant. MFE—Minimum Free Energy.

The *in silico* analysis using RNAfold revealed that the existence of the T variant in position 402 of the 3’UTR region alters the minimum free energy (MFE) of 1B and 1C mRNA transcripts from -973.70 kcal/mol to -975.30kcal/mol for 1B transcript and from -875.20kcal/mol to -877.20kcal/mol for 1C transcript. The MFE for the 1A transcript is negligible after 3’UTR modification in position 402 by the existence of the C or T variant (-857.90kcal/mol to -857.20kcal/mol). Moreover, the RNAfold results strongly suggest that the C/T substitution has a marked effect on the secondary structure of all studied *NEMO* gene transcripts (Fig 1).

Additional *in silico* analyses conducted by RegRNA and miRNA_target software pointed out that the existence of the variant T in position 402 of the 3’UTR region may modify the binding site for miRNA. The results suggest that when cytosine is in position 402 of the 3’UTR, it creates potential binding sites for miR-3960 and miR-5090, whereas when thymidine is in this position, it creates potential sites for miR-503, miR-885-3p, miR-1266 and miR-4519.

## Discussion

This is the first study to identify a relationship between the simultaneous occurrence of identical variant of the *NEMO* gene in the maternal and fetal genomes, and a higher likelihood of preeclampsia development. NEMO is an essential regulator of NFĸB activation [15]. This transcription factor is indirectly implicated in the regulation of many processes related to immunological responses, and cell differentiation or apoptosis. Upon NFĸB activation, it is translocated into the nucleus, where it regulates the expression of genes which code cytokines, interleukins, growth factors and adhesion molecules [19,32]. Most of these factors are perceived as markers of preeclampsia [33]. A study conducted by Luppi et al [34] reveals that the level of NFĸB is significantly higher in the blood of preeclamptic women than in non-complicated gestations. This may be the main reason for the observed elevated levels of IL-1, IL-6 and TNFα, whose genes are directly under the control of NFĸB activated through the classical NEMO-dependent pathway [34]. The elevated level of NFĸB in the blood of preeclamptic women observed by other researchers correlates positively with the results of our recent studies, in which a higher level of *NEMO* gene expression was observed in the blood of women whose gestation was complicated by hypertension and proteinuria [25].

Until now, studies into the correlation between the presence of various genetic markers and the development of preeclampsia were conducted generally among populations of pregnant women. In this study, we propose a different perspective on the genetic predisposition of preeclampsia. A significant body of evidence from literature studies has highlighted the following five key points: (1) preeclampsia is inherited through maternal lineage, (2) expression of the described disorder may differ between two pregnancies undergone by the same woman, (3) fetal (paternal) genes are implicated in preeclampsia development, (4) a male fetus is much more common in preeclampsia and (5) the level of *NEMO* gene expression is higher in blood of preeclamptic women and their children [3,4,6–8,25]. Hence, we propose that the presence of the same variant of the *NEMO* gene in the maternal and fetal genomes (chromosome Xq28) may increase the likelihood of preeclampsia development.

During *NEMO* gene sequencing of material from preeclamptic mothers, two single nucleotide variants were identified: IKBKG:c.*368C>A and IKBKG:c.*402C>T, both localized in the 3'UTR region. The frequency of occurrence of these variants was assessed in the DNA of fetuses qualified for the control and study groups, and in DNA derived from preeclamptic mothers and women who had experienced non-complicated pregnancy. Our analyses reveal that the simultaneous occurrence of a maternal TT genotype and a TT genotype in the daughter, or a T allele in the son, increased the risk of preeclampsia development (OR 2.59, 95%Cl 1.20–5.57; p<0.05).

Of course we acknowledge that preeclampsia is a multifactorial disorder and that no single genetic marker causes preeclampsia development, but the marker may modulate the predisposition to preeclampsia. It is highly probable that other genetic and environmental factors exist, whose combination is implicated in preeclampsia development [3,4,35,36]. We suppose that the maternal/fetal carriage of IKBKG:c.*402T variant may be responsible for activating one of the numerous pathways of preeclampsia development. For this reason, the maternal TT/fetal TT or T genotype is also observed (although significantly decreased) in non-complicated pregnancies.

Little is known of the possible mechanism of action of the rs782437119 variation. As was mentioned above, this variation is localised in the 3’UTR region of the NEMO gene. The sequence of elements within mRNA determines its half-life. Some sequences promote rapid mRNA decay while others promote mRNA stabilization [37]. To find the hypothetical mechanism of influence of the IKBKG:c.*402C>T variant on the half-life of mRNA, bioinformatic analyses were conducted. The first analysis was carried out using RNAfold software to determine whether some differences exist between the minimum free energy (MFE) of the secondary structure of IKBKG:c.*402C and IKBKG:c.*402T mRNA variants which may influence the stability of this particle. This analysis reveals that when the transition from C to T in the 402^nd^ position of the 3’UTR region is observed, the minimum free energy of 1B and 1C transcripts is decreased: the fold change for 1B is -1.6, whereas for 1C is -2.0. Although the magnitudes of change of MFE for 1B and 1C transcripts are small, it is possible that the observed transition may in some way influence the length of the half-life of these transcripts, especially for transcript 1C. Some *in silico* and *in vitro* studies present that the difference (fold change about 2.0) in the MFE for two transcripts of the same gene plays a significant role in the observed difference in their stability [38,39]. Moreover, bioinformatics analyses conducted in this study show that the absence of the T nucleotide in position 402 influence the secondary structure of mRNA for all transcripts. The change in the secondary structure of mRNA may cause that this structure is unlikely to provide the required specificity for many RNA binding proteins (RBPs) which are responsible for post-transcriptional regulation of mRNA stability, translation, cell localisation and splicing [40].

To find the relationships between *NEMO* gene expression in placentas, maternal and fetal blood and the simultaneous carriage of IKBKG:c.*402 TT genotype by the mother, and the TT genotype or T allele by her child, the study and control groups were merged together and then separated into two subgroups according to their set of genotypes. The first (mutated) subgroup included preeclamptic and control cases (mother TT/daughter TT or mother TT/son T) whereas the second (non-mutated) group included cases whose genotypes included at least one C allele (IKBKG:c.*402C/T).

The statistical analyses conducted among these subgroups reveal that all transcripts, especially 1C, are more plentiful in maternal blood and umbilical cord blood in the mutated group: mother TT/daughter TT or mother TT/son T. However, only the 1A transcript in maternal blood and the Total NEMO, 1A and 1C transcripts in umbilical cord blood were found to be significantly different between the mutated and non-mutated groups. The same analyses revealed that no discrepancy in placental level of *NEMO* gene expression was observed between the two mutated and non-mutated subgroups.

With regard to the set of maternal/fetal genotypes, the discrepancy observed for blood and placental *NEMO* gene expression may be related to the different etiologies behind the activation of the preeclamptic pathway in placentas and in blood cells. However, to elucidate this phenomenon further research is required to gain a more concrete idea of the complex relationships associated with the 3’UTR *NEMO* gene variation and its influence on blood and placental tissue.

Because the stability of mRNA not only depends on the free energy of its secondary structure but also from other factors such as its affinity to degradation by miRNA, further analyses using RegRNA2 and miRNA_target were conducted. These analyses revealed that the target site for several different miRNA appeared when a thymine (T) is present in the position 402 of 3’UTR of the *NEMO* gene. Strong interaction was found between miR-503,-885-3p,-1266 and 4519, and the mRNA of the *NEMO* gene for allele T. To confirm the results of these bioinformatic analyses, additional functional studies are warranted in the future.

To the best of our knowledge, no previous study has examined the level of miR-885-3p, miR-1266 and miR-4519 in non-complicated or pathophysiological gestations. Only miR-503 was studied in serum and placentas of expected mothers with and without preeclampsia. The microarray study conducted by Akehurst et al [41] reveals that miR-503 increases in plasma of pregnant women. Moreover, microarray analysis shows differential miRNA-503 level in plasma between women who develop late preeclampsia and normotensive controls. The level of miR-503 was decreased (although not significantly) in plasma of preeclamptic women at 28^th^ week of gestation [41].

Mouillet et al [42] study shows that hypoxia in primary human trophoblast cells lead to abrogation of miR-503 level. This observation suggests that also in preeclamptic placentas in which hypoxia dominates the level of miR-503 should be also depleted. An additional information about miR-503 and preeclampsia is given by the microarray analyses presented in GEO Profiles database (http://www.ncbi.nlm.nih.gov/geoprofiles/?term=miR503+preecalmspia). The GDS2080 examination conducted among 14 samples (4 controls, 5 early and 5 late preeclampsia) shows that both in early and late preeclamptic placentas, the level of miR-503 was negligible decreased compared to controls. Moreover, the GDS2548 and GDS3457 studies pointed out very similar placental level of miR-503 (during the first and the third trimesters) in preeclamptic and non-complicated gestations. Lack of significant discrepancy in miR-503 level both between preclamptic and normotensive and between preeclamptic and IUGR placentas was also presented by Guo et al.[43].

We acknowledge that our study has some limitations: first we focus only on the maternal exome of the *NEMO* gene. The expansion of the sequencing process to the IKBKG maternal introns and to the entire IKBKG fetal gene may deliver further interesting information. This new knowledge may be helpful in determining why the level of *NEMO* gene expression is significantly decreased in preeclamptic placentas in comparison to non-complicated pregnancies. Secondly, the study is based on *in silico* studies rather than laboratory studies, which may explain how the IKBKG:c*402C/T variant influences the regulation of *NEMO* gene expression in maternal and fetal blood and whether the appearance of a maternal TT/fetal TT or T genotype may in some way be responsible for regulation of *NEMO* gene expression in placental cells. It is obvious that the same variation may give various effects dependent on the type of tissue [44]. Thirdly, our study was not extended to identify other mechanisms which may influence the regulation of *NEMO* gene expression e.g. profile of methylation of the CpG island present in the promoter region of the *NEMO* gene [45].

In summary, this is the first study to report an association between single nucleotide variant in *NEMO* gene (IKBKG:c.*402C>T) and the elevated risk of preeclampsia development. The simultaneous occurrence of a TT genotype in the mother and daughter, or a T allele in her son, was associated with a 2.59-fold increase in the risk of hypertension and proteinuria appearance during gestation. Moreover, we have shown that maternal and fetal genotype correlated with the level of *NEMO* gene expression in their blood. Future examinations should be conducted to determine how the IKBKG:c.*402C>T variant influences the development of preeclampsia. Our observations may offer a new insight into the genetic etiology and pathogenesis of preeclampsia.

## Supporting information

S1 File Photography of distribution bands on polyacrylamide gel for IKBKG:c.*368C>A and IKBKG:c.*402C>T variants. (PDF)

S1 Table Maternal and fetal genotypes. (XLSX)

S2 Table Comparison of *NEMO* gene expression in umbilical cord blood of preeclamptic and control children. (DOCX)
