# Supplementary material for: Double hit of NEMO gene in preeclampsia
Source: PLoS One. 2017 Jun 27;12(6):e0180065. doi: 10.1371/journal.pone.0180065 (PMC5487068; doi:10.1371/journal.pone.0180065)
Supplement: S1 File — (PDF) [file pone.0180065.s001.pdf]

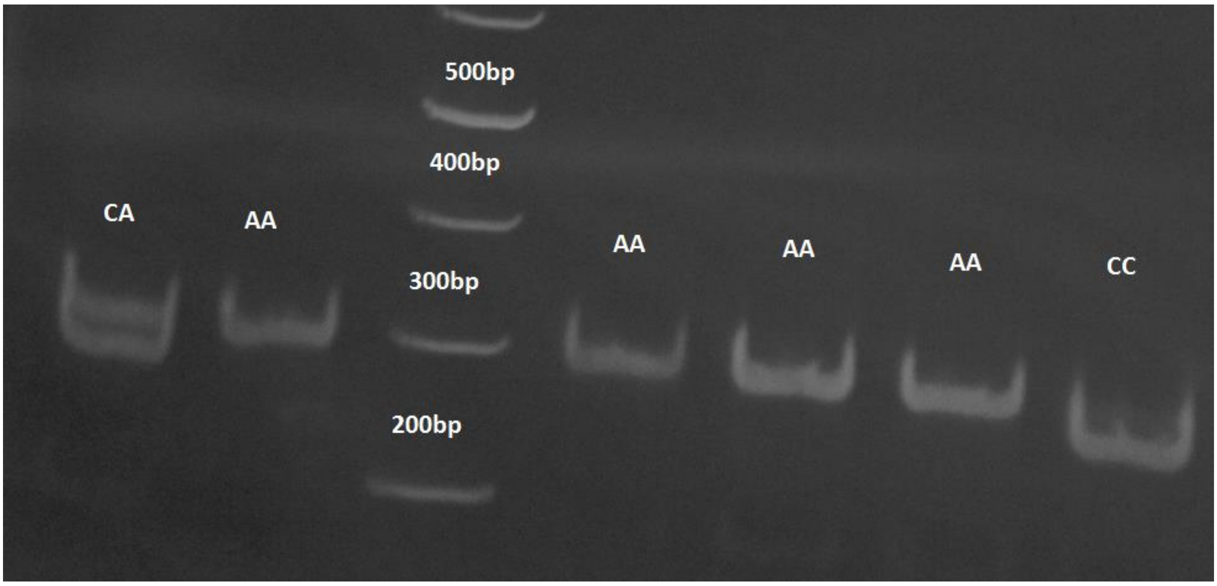

Fot.1 Bands distribution on polyacrylamide gel for IKBKG:c.*368C>T variant. The observed genotypes: CC-269bp; CA-269bp+292bp; AA-292bp

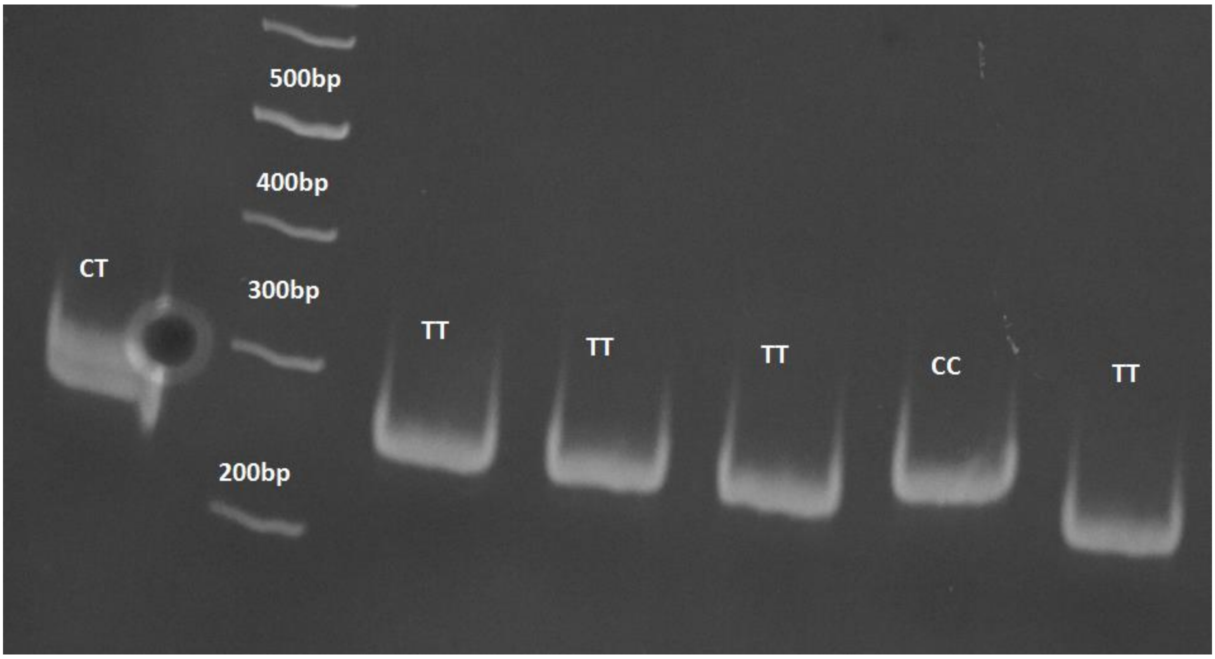

Fot.2 Bands distribution on polyacrylamide gel for IKBKG:c.*402C>T (rs782437119) variant. The observed genotypes: CC-270bp; CT-270bp+252bp; TT-252bp
