# Supplementary material for: Double hit of NEMO gene in preeclampsia
Source: PLoS One. 2017 Jun 27;12(6):e0180065. doi: 10.1371/journal.pone.0180065 (PMC5487068; doi:10.1371/journal.pone.0180065)
Supplement: S2 Table — (DOCX) [file pone.0180065.s003.docx]

| Transcript | Preeclmapsia  median  (interquartile range) | Control  median  (interquartile range) | p^a^ |
| --- | --- | --- | --- |
| Total NEMO | 3.83  (1.69 – 11.21) | 3.86  (1.14 – 8.50) | 0.321 |
| 1A | 1.61  (0.50 – 3.44) | 0.60  (0.24 – 1.42) | <0.001 |
| 1B | 1.18  (0.27-3.30) | 0.77  (0.19 – 1.69) | <0.05 |
| 1C | 1.78  (0.15 – 10.61) | 0.75  (0.03 – 2.27) | <0.01 |

Table 2. Comparison of *NEMO* gene expression in umbilical cord blood of preeclamptic and control children

The values are presented as relative gene expression levels calculated by the use of Pfaffl method

^a^ – p value was calculated by the use of the Mann-Whitney U test
